# Large Vestibular Aqueduct-Associated Symptoms: Endolymphatic Duct Blockage as a Surgical Treatment

**DOI:** 10.3390/audiolres14020027

**Published:** 2024-03-18

**Authors:** Issam Saliba, Sarah Alshehri, Isabelle Fournier, Nasser Altamami

**Affiliations:** 1 Division of Otorhinolaryngology and Head & Neck Surgery, University of Montreal, Montreal, QC H3C 3J7, Canada; 2 University of Montreal Health Center (CHUM), Department of Otorhinolaryngology and Head & Neck Surgery, Montreal, QC H2X 3E4, Canada; 3 University of Montreal Health Center Research Center (CRCHUM), Montreal, QC H2X 0A9, Canada; 4 Sainte-Justine University Hospital Center (CHU-SJ), Otorhinolaryngology and Head & Neck Surgery, Montreal, QC H3T 1C5, Canada

**Keywords:** vestibular aqueduct, enlarged, large, duct blockage, endolymphatic, hearing loss, head trauma, third window, Ménière

## Abstract

Objective: This study aimed to evaluate the effectiveness of endolymphatic duct blockage (EDB) on dizziness control in patients with a large vestibular aqueduct (LVA) and to evaluate its effect on hearing. Study design: This is a prospective nonrandomized study. Setting: Five adults and one child with dizziness and five children with progressive hearing loss were referred to our tertiary centers. Methods: The dizziness handicap inventory (DHI) and DHI-PC (dizziness handicap inventory—patient caregiver) questionnaires were used before and after surgery. All patients underwent a preoperative temporal bone HRCT scan and pure tone audiometry one day before surgery, then four and twelve months after surgery and at the last follow-up. The mean follow-up time was 5.6 years. Student’s *t*-test was used to compare DHI/-PC results. Results: The DHI scores were 44, 24, 84, 59 and 56 before surgery, respectively, for Patients 1 to 5. The DHI scores at four months was significantly different, i.e., 4, 6, 0, 7 and 18 (*p* = 0.001). No differences were found between 4 and 12 months. Patient 6 (child) had Trisomy 21; their DHI-PC score dropped from 38 (preoperative score) to 8 (postoperative score), showing no activity limitations; clinical evaluation showed the complete resolution of symptoms. We found no significant differences between hearing loss before the surgery and at 1 and 12 months post operation for four adult patients. Our fifth adult patient’s hearing changed from severe to profound SNHL. For 5 out of 6 pediatric patients, preoperative PTA and mean ABG were 63 dB and 20 dB, respectively; postoperatively, they improved to 42 dB and 16 dB, respectively. The hearing loss level for the sixth pediatric patient dropped from moderate (PTA = 42 dB) to severe (PTA = 85 dB) due to an opening of the endolymphatic sac and a sudden leak of the endolymph. Conclusions: EDB, using two titanium clips, seems to be helpful for controlling vestibular symptoms and for stabilizing hearing or even to improve hearing in 82% of cases. Nevertheless, there is a risk of hearing worsening.

## 1. Introduction

The vestibular aqueduct is a bony canal that runs between the vestibule medially and the petrous temporal bone posteriorly. This inverted J shape of the vestibular aqueduct is the result of downward traction on the distal endolymphatic system that continues even after the otic labyrinth has reached its normal adult form at midterm [1]. The endolymphatic membranous duct passes through the bony vestibular aqueduct to reach the endolymphatic sac [2].

The large vestibular aqueduct (LVA) is a congenital malformation that predisposes individuals to variable vestibulo-cochlear symptoms. The first description of LVA was reported by Carlo Mondini in 1791 during the temporal bone dissection of a child who had congenital sensorineural hearing loss [3]. According to Pyle, LVA may be the result of continued aberrant growth rather than a failure of shrinkage or developmental arrest early in embryogenesis [4]. This temporal bone anatomical abnormality includes the duct and the endolymphatic sac enlargement. A large vestibular aqueduct is the most common congenital deformity, found in the inner ear tomography of 1 to 1.5% of the population [2,4,5].

LVA is diagnosed by a temporal bone high resolution computed tomography (HRCT) scan. In 1978, Valvassori and Clemis reported the first clinical case of LVA; from this point on, the vestibular aqueduct has been considered enlarged if the axial plane of the CT scan shows a width of at least 1.5 mm at the midpoint and 2.0 mm at the operculum [2,3,4,5,6,7,8,9]. 

In a recent study performed by our group of over 1812 temporal bone CT scans, 101 temporal bones were found to have LVA; in few cases, axial CT scan cuts are not conclusive. In 2012, our team reported an alternative measure to diagnose LVA by using CT scans of coronal cuts: a width larger than 2.4 mm at the midpoint and/or 4.4 mm at the operculum is considered positive for LVA [5]. 

With LVA, hearing levels can fluctuate or progressively deteriorate, therefore, hearing loss can change throughout life [10]. However, according to Leveson et al., hearing loss in children with LVA is acquired during childhood [10]. Although LVA accounts for approximately 15% of pediatric sensorineural hearing loss (SNHL) cases and is present in a heterogenous population of patients, the progression of HL may or may not happen [11]. 

Many reports of LVA have discussed its clinical presentation and have focused on the hearing loss related to this pathology. The mechanism of hearing loss associated with this anatomical anomaly is still not very clear. However, individuals with LVA may also experience episodic vertigo or dizziness that can affect their quality of life [6]. Only a few studies have focused on vertigo in patients with LVA, and no previous reports have discussed the prevalence or the management of vestibular symptoms caused by LVA. Patients with LVA should be cautioned of the risk of developing a vestibular pathology. To date, no surgical treatment has been proposed to relieve these symptoms.

When the volume of the vestibular aqueduct and endolymphatic sac increases, it causes structural, auditory and vestibular physiologies and mechanism anomalies. In these cases, auditory data have been more frequently studied and analyzed in the literature than vestibular complaints. To our knowledge, few descriptions of such vestibular signs and symptoms are extant in the literature; existing reports range from mild instabilities to real, episodic vertigo [6]. This discrepancy in patients with LVA is likely due to small sample sizes, different follow-up periods, ascertainment bias due to recruitment method subjects or a combination of these factors. Vestibular signs and symptoms include a mixture of true rotational vertigo, dizziness, head tilting with vomiting at a prelingual age and/or delayed motor development.

Different surgical attempts have been reported in the literature, like endolymphatic sac surgery, endolymphatic subarachnoid shunting and LVA occlusion; none of these trials yielded satisfactory results and were limited by postoperative hearing loss.

Endolymphatic duct blockage (EDB) surgery was described in 2015 by our group as a novel treatment for refractory Ménière disease. It controls symptoms with significant success, supported by 12 years of experience; the technique of EDB is similar to endolymphatic sac decompression but it is completed by a crucial step: blocking the endolymphatic duct using two titanium clips [12]. Although EDB is effective, it is not a destructive technique: there is no clinical cochlear or vestibular damage. There is significantly better control of vertigo attacks compared to traditional endolymphatic sac decompression for Ménière’s disease [12]. Using EDB to treat symptomatic LVA by decreasing the width of the endolymphatic duct or by totally blocking the route between the endolymphatic sac and the inner ear could be an option. We hypothesize that pressure regulation may mitigate inner ear damage.

Therefore, this study aims to evaluate the effect of endolymphatic duct blockage on dizziness control and hearing preservation in patients with LVA.

## 2. Methods

### 2.1. Study Design

This prospective nonrandomized study, conducted between 2015 and 2022, comprised 11 patients. Our inclusion criteria included adult or pediatric patients with LVA who were suffering from dizziness or vertigo that affected their daily lives and/or who were experiencing progressive hearing loss due to LVA. Exclusion criteria include other causes of dizziness like, but not limited, to vestibular migraines, Ménière’s disease, persistent perceptual postural dizziness (PPPD) or benign paroxysmal positional vertigo (BPPV). Five adult and six child patients were referred to our otology and neurotology adult or pediatric tertiary centers for the management of symptoms related to LVA. 

The average age for patients suffering from progressive hearing loss was 4.06 ± 0.99 years (all children) and 30.41 ± 16.13 years for patients suffering from dizziness (5 adults and 1 child). The duration between the diagnoses and the EDB was 5.5 ± 2.17 years for hearing loss and 3.5 ± 1.82 years for dizziness.

All adult patients suffered from vestibular symptoms associated with LVA with stable, preserved functional hearing levels. The patients suffered mainly from dizziness described as a constant instability rather than rotatory vertigo, which affected their daily activities. Five out of six pediatric patients reported progressive mixed hearing loss related to LVA without dizziness or vertigo. 

The sixth pediatric patient, known to have Trisomy 21, was followed-up by his pediatric otolaryngologist for progressive bilateral mixed hearing loss since the age of two years. By the age of 12 years, he had developed episodic dizziness affecting his daily activities; therefore, he was referred to our clinic. 

The five adult patients suffering from dizziness received the dizziness handicap inventory (DHI) questionnaire electronically for an evaluation of the severity of dizziness symptoms before and after surgery (Table S1). Only one pediatric patient suffering from dizziness received the dizziness handicap inventory for patient caregiver (DHI-PC) questionnaire electronically to assess the severity of dizziness symptoms before and after surgery (Table S2). Because the other patients did not suffer from imbalance, the questionnaire was not sent to them.

DHI is a validated questionnaire and is one of the most used questionnaires to evaluate the impact of vertigo on the quality of life [13]; it consists of 25 questions designed to incorporate functional (F), physical (P) and emotional (E) impacts on the quality of life. The following scores can be assigned to each item: No = 0; Sometimes = 2; and Yes = 4. The final score is then calculated. The maximum score is 100 (36 points for functional, 36 points for emotional and 28 points for physical) and the minimum score is 0.

The final score determines the severity of the disability related to vertigo which is classified as mild disability (16 to 34 points), moderate disability (36 to 52 points), and severe disability (54 and more points).

The DHI-PC represents a new tool for evaluating the effect of pediatric dizziness on patients between the ages of 5 and 12 years of age (as viewed through the perspective of the caregiver). DHI-PC is reliable, does not take much time to perform and is easy to analyze. It is a patient-reported inventory that quantifies dizziness-related symptoms and the associated disability experienced by the child. The questionnaire is designed to be completed by the caregiver and is used to identify the difficulties and impact of dizziness on the child’s life. The DHI-PC is considered a valuable tool for screening and follow-up in the management of pediatric dizziness.

The DHI-PC^14^ has 21 questions developed for parents or caregivers to report their children’s dizziness symptoms. The aim is to have a better understanding of how dizziness or balance issues affect their lives. Some examples of questions include the following: Does your child’s problem make him or her feel tired? Is your child’s life ruled by his or her problem? Does your child’s problem make it difficult for him or her to play? The responses are scored from “yes” (4 points), “sometimes” (2 points) or “no” (scored as 0). The DHI-PC total score of 0 to 16 is classified as no activity limitations. A score of 16 to 26 indicates mild activity limitations, a score of 26 to 43 indicates moderate limitations and a score of more than 43 indicates severe limitations. The DHI-PC instrument has high internal consistency for the total scale (Cronbach’s a = 0.93) and a high test–retest reliability (r = 0.98, *p* ≤ 0.001) [14].

Given the fact that our aim was to purely evaluate the subjective outcomes of EDB on dizziness control, the objective data obtained from sources such as videonystagmogram, the video head impulse test (vHIT), and vestibular evoked myogenic potential (VEMP) were not considered in this study. 

All patients underwent a temporal HRCT scan and were clinically evaluated. In this study, for adult or pediatric patients, we followed the Valvassori definition for the diagnosis of LVA using axial cuts of the CT scan: a diameter more than 1.5 mm at the midpoint or greater than 2.0 mm at the operculum were considered positive [2,3,4,5].

Pure tone audiometry was carried out for all included patients one day before surgery and four months, twelve months and at the last follow-up postoperatively. Dizziness symptoms were evaluated using the DHI and DHI-PC before surgery and at four and twelve months postoperatively.

All adult patients and the parents of pediatric patients signed an informed consent regarding the surgery and its possible risks and complications. In addition, patients and parents were made explicitly aware that this surgery is experimental for this indication and is associated with a tangible risk of worsened hearing loss. They were informed that the same procedure is performed for patients with refractory Ménière’s disease with highly positive results.

The study was approved by our institutional research ethics board and follows the standards of our institutional ethical committee. All survey answers were analyzed anonymously and confidentially.

### 2.2. Statistical Analyses

Student’s *t*-test was used to compare the DHI results. A *p* value of lower than 0.05 was considered statistically significant.

### 2.3. Surgical Protocol of EDB

**Figure 1:**
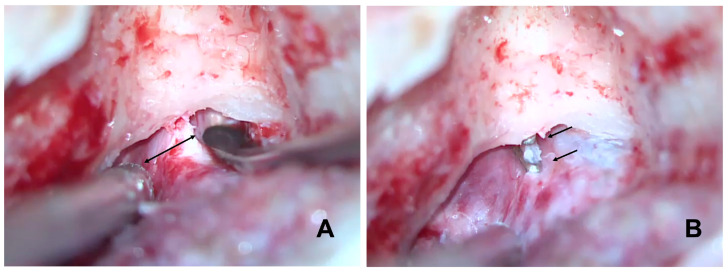
Shows the enlarged endolymphatic duct intraoperatively: the double-headed arrow indicates the width of the enlarged endolymphatic duct (**A**) in a left ear, the two arrows show the 2 titanium clips totally blocking the enlarged duct (**B**). The spatula and the suction (**A**) retract the dura mater from either side of the enlarged endolymphatic duct to improve visibility.

Canal wall-up mastoidectomy was performed as follows. The identification of the tegmen mastoideum, sigmoid sinus and sinodural angle was required. The posterior bony wall of the external ear canal was thinned. We identified the posterior semicircular canal and the dura matter of the posterior fossa. Using the prominence of the horizontal semicircular canal, the Donaldson line was identified to approximate the position of the endolymphatic sac. The bone over the dura of the posterior fossa was thinned with diamond burrs. The sac was completely skeletonized. Special attention should be paid to this section. LVA makes the EDB procedure more difficult as it has higher risks than EDB performed for Ménière’s disease. The enlarged sac is very thin and larger in size. The endolymphatic pressure inside the sac makes the possibility of tearing and even the explosion of the sac during the dissection more likely. The infralabyrinthine dura was exposed because the main body of the sac and its lumen often lie within this area. The sac was neither incised nor removed from the posterior fossa dura. The dissection of the bone around the vestibular aqueduct operculum and the posterior fossa dura was completed. The dissection continued both superiorly and inferiorly in order to identify the endolymphatic duct in its superior and inferior locations in continuity with the endolymphatic sac to create sufficient space to insert the tips of the crimper and to clip the duct. Finally, we blocked the dissected endolymphatic duct with two small titanium clips (Figure 1).

B: identifies the enlarged endolymphatic duct before its entrance into the labyrinthine bone.

**Figure 2:**
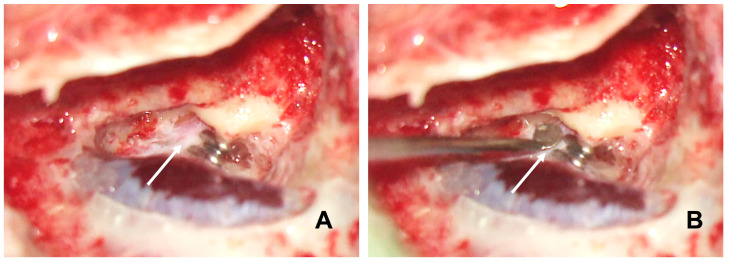
Shows the enlarged endolymphatic duct partially blocked by the titanium clips in a left ear intraoperatively (**A**). The arrow indicates the remaining unblocked part of the enlarged endolymphatic duct. The size of the remaining unblocked duct was estimated using a 2 mm Rosen ear knife (arrow) (**B**).

The titanium clips were applied using the ligating clip applier, which is similar to the clip used in vascular surgery. Often, the enlarged duct was larger than the titanium clip, making total duct clipping impossible. Partially clipping the enlarged duct can reduce it to a normal size range (Figure 2); in fact, we think it is unnecessary to block it completely like we do in the EDB treatment for Ménière’s disease. However, the large clip could be an option to totally block the duct in case enough space is available to insert a large clip applier. Finally, we filled the mastoid cavity with bone dust collected throughout the mastoidectomy. All patients were discharged from the hospital the same day after surgery.

Follow-up was performed at 1 week, 4 and 12 months and then every year postoperatively.

## 3. Results

Eleven patients (5 adults and 6 children) with LVA were included in our study (4 males and 7 females). Only Patient 5 presented with bilateral LVA and was operated on only on the larger side (left). LVA was found on the right side in six patients, and on the left side on the remaining five patients. The average of age of the adults was 37.74 ± 15.06 years, and it was 10.3 ± 2.55 years for the children (Table 1). Patients 3 and 10 were the only patients who had a partial EDB blockage.

No patient was noted to have SLC26A4A mutations and/or Pendred’s associated disease. In addition, no patient had concurrent cochlear dysplasia like an incomplete partition type 2 anomaly, which is common in patients with LVA.

### 3.1. Vestibular Evaluation

**Figure 3:**
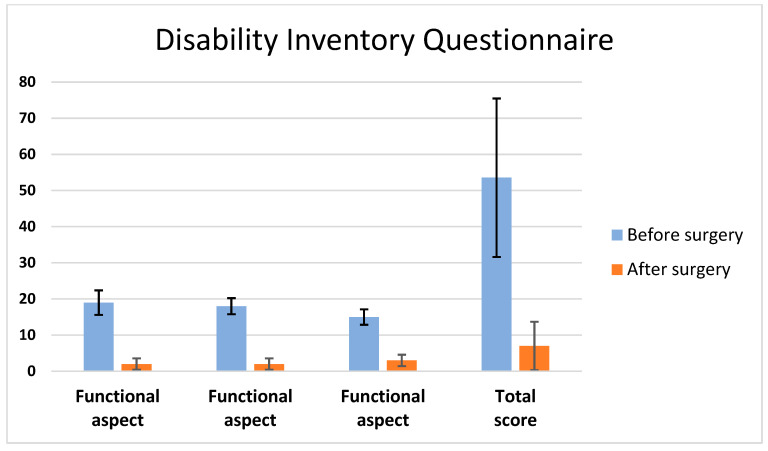
The dizziness handicap inventory questionnaire scores significantly changed before and after surgery, reflecting the improvement of dizziness symptoms after the blockage of the endolymphatic canal at four months postoperatively (Patients 1 to 5).

Patients 1 to 6 had presented with dizziness symptoms with episodic instability rather than rotatory vertigo 2 to 3 years before their arrival at our clinic. Neurotological examination revealed signs of unilateral vestibular dysfunction: the Fukuda and head-thrust tests were positive on the affected side. The dizziness symptoms became constant and severe enough to affect their daily activities. We used the dizziness handicap inventory questionnaire to evaluate the severity of dizziness before surgery. The total scores of handicaps due to dizziness were 44, 24, 84, 59 and 56 before surgery, respectively, for Patients 1 to 5. Four months after surgery, the DHI was used to evaluate their improvement. The scores had changed significantly, i.e., 4, 6, 0, 7 and 18, respectively, for Patients 1 to 5 (*p* = 0.001) (Figure 3). The last patient noticed a progressive but slow improvement of their instability symptoms. No statistically significant differences were found at 12 months compared to 4 months postoperatively on the DHI score (*p* > 0.05).

All patients were free of other medical illnesses except Patient 6 who is a 14-year-old male known to have a Trisomy 21. This patient had unilateral progressive mixed hearing loss diagnosed at the age of two years. He developed episodic dizziness at the age of ten years, which progressively increased in severity. The DHI-PC scoring was applicable for this patient. Total disability score due to dizziness was 38 before surgery, indicating moderate activity limitations. One month after surgery, this patient noticed a progressive and gradual improvement of his dizziness. Four months after surgery, the DHI-PC was used to evaluate improvement. The score had dropped from 38 to 8, showing no activity limitations. However, the overall evaluation carried out by his parents in addition to the clinical neurotological examination showed a complete resolution of his vestibular symptoms. The mean follow-up time was 5.6 years. No recurrence of symptoms were noticed at the time of the last visit.

### 3.2. Hearing Level

All patients had mixed hearing loss with the air–bone gap mainly affecting low frequencies and severe sensorineural hearing loss affecting mainly high frequencies due to a large vestibular aqueduct. 

**Figure 4:**
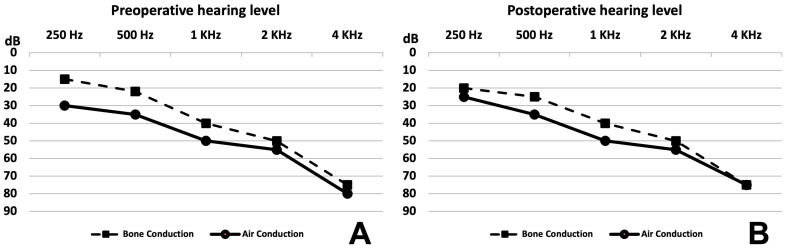
Adult hearing levels. (**A**) Mean hearing level of Patients 1, 2, 3 and 4 before surgery showing mixed hearing loss (air–bone gap average: 9.6 dB). (**B**) Mean hearing level of Patients 1, 2, 3 and 4 at 12 months after surgery showing mixed hearing loss (mild to moderate) and an air–bone gap average of 6 dB.

There were no differences in the averages of the hearing loss level before surgery and 4 and 12 months after surgery for the four adult patients (Figure 4). Details of hearing thresholds are reported in Table 2. However, the fifth adult patient had severe hearing loss before surgery, which dropped to profound hearing loss at day 1 post operation with no improvement despite medical treatment with oral steroid and intratympanic steroid injection. No unusual manipulation or intraoperative complications occurred for the fifth patient.

**Figure 5:**
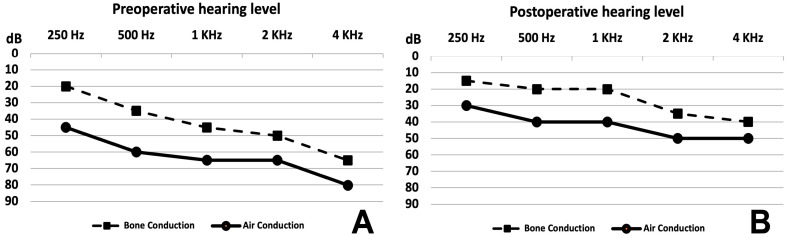
Pediatric hearing levels. (**A**) Mean hearing level of Patients 6, 7, 8, 9 and 11 before surgery showing mixed hearing loss (air–bone gap average: 20 dB). (**B**) Mean hearing level of Patients 6, 7, 8, 9 and 11 at 12 months after surgery showing mixed hearing loss (mild to moderate) and air–bone gap average of 16 dB.

The average hearing level surprisingly improved after surgery in four and remained stable in one out of the six pediatric patients, as shown in Figure 5A (before surgery) and Figure 5B (after surgery). Improvement was statistically significant for bone and air conduction. Details are provided in Table 2. Before surgery, the pure-tone average (PTA) of air conduction and the mean air–bone gap (ABG) at frequencies from 250 Hz to 4 KHz were 63 dB and 20 dB, respectively. After surgery, the PTA and the mean ABG at all frequencies were 42 dB and 16 dB, respectively. Clinically, hearing remained stable as of the most recent follow-up.

However, Patient 10 developed profound hearing loss postoperatively. Intraoperatively, the endolymphatic sac of Patient 10 exploded despite all meticulous and smooth dissection. The membrane was very thin, transparent and bulging, reflecting possible high endolymphatic pressure inside the sac. Therefore, the hearing level of the Patient 10 dropped from moderate (PTA = 42 dB) to profound (PTA = 85 dB) hearing loss postoperatively with no improvement despite oral steroid treatment. The parents preferred not to perform intratympanic steroid injection. Thus, it was not included in the mean hearing level follow-up in the 12th month post operation because we aimed to evaluate the stability of preserved hearing at one year after surgery. 

At the mean follow-up time of 5.6 years, no changes were noted in the hearing tests when compared to the tests performed at 12 months post operation. 

### 3.3. CT Scan

**Figure 6:**
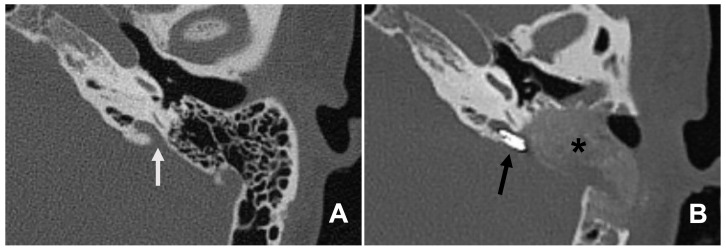
(**A**) Left large vestibular aqueduct (LVA) (white arrow) and (**B**) same LVA at day 1 after surgery with two titanium clips (black arrow) blocking the endolymphatic duct placed at the level of the vestibular aqueduct operculum. * Shows bone dust filling the mastoid cavity.

The diagnosis of all patients with LVA was confirmed by a HRCT scan. Figure 6 shows a HRCT scan of a left-side LVA before and after clipping. One patient presented a bilateral LVA (Patient 5) and was operated only on the larger side, which was the left side. The vestibular aqueduct diameter was measured on an axial cut of the HRCT scan and was found to range from 1.97 to 4.26 mm at the midpoint and from 2.85 to 6.46 mm at the operculum. The average sizes at the midpoint and at the operculum were 2.78 ± 0.4 (confidence interval) and 4.36 ± 1.12 mm (confidence interval), respectively. 

### 3.4. Partial Duct Blocking

Patients 3 and 10 had partial EDB. However, symptom improvement was similar to those with a complete duct blockage and hearing remained stable as of the last follow-up in comparison to the tests performed at 12 months post operation.

## 4. Discussion

The pathophysiology of vestibular symptoms associated with LVA is poorly understood and not widely discussed. The impact of these symptoms on the quality of a patient’s life is severe and might affect their daily activities as a functional, physical and emotional disability, as shown in our study from the dizziness handicap inventory questionnaire. 

Different hypotheses have been proposed to explain the hearing loss experienced by patients presenting LVA. Head trauma may lead to intracochlear membrane rupture, and the admixture of the endolymph with the perilymph may be the cause behind sudden hearing loss following head trauma [9]. A widely dilated patent endolymphatic duct is at risk of the reflux of the hyperosmolar endolymphatic sac’s contents, as described by Levenson et al. [10]. About one in three people with LVA present with sudden hearing loss after a minor head injury or barotrauma. The risk seems to be higher in people experiencing severe hearing fluctuations [15]. People with LVA are advised to protect their hearing by avoiding contact sports and all risk of barotrauma. They should wear head protection equipment such as a helmet for activities like bike riding or skiing and to use a seat belt whenever this is suggested. Considering the same mechanism for vestibular disturbance, then decreasing the pressure and the reflux of the endolymph from the sac to the labyrinth by clipping the endolymphatic duct could be a preventive solution of further inner ear damage.

On the other hand, a meta-analysis was performed in 2015 by Alemi et al., which reported long-term progressive sensorineural hearing loss as a common complication of large vestibular aqueduct; however, the association with head trauma is not strongly supported [16].

In addition, Brodsky et al. recommended in their literature review in 2018 that, due to the absence of enough evidence, physicians should limit patients from playing contact sports due to LVA alone [17]. Faced with these reported data, it seems that the literature is somewhat confusing. According to our records, few patients developed hearing loss after a minor head trauma. Therefore, we believe that head trauma could be problematic, and we completely agree with Levenson et al. and Stahl et al.; we suggest that our non-operated LVA patients protect themselves from head trauma by avoiding contact sports and all risk of barotrauma. 

There is no agreement or clear protocol on the management of severe symptoms related to LVA. No published trial supports the treatment of sudden hearing loss in LVA with corticosteroids. Steroid administration was not reported in any of the studies included in the literature review published in 2018 [17]. The surgical treatment of large vestibular aqueduct has been attempted However, all trials ended with non-satisfactory results; therefore, surgical management was no longer recommended for these cases. A retrospective analysis published in 1988 by Jackler et al. demonstrated that 50% of patients with congenital progressive hearing loss presented with an LVA [7]. They demonstrated no benefit of endolymphatic sac surgery; thus, they did not recommend endolymphatic sac surgery for hearing preservation as a surgical treatment of LVA [7]. In 1989, once again, Jackler and De La Cruz reported their results on endolymphatic subarachnoid shunting performed on seven patients suffering from LVA to stabilize hearing. Four of these ears had a significant immediate postoperative drop in hearing. For this reason, endolymphatic sac shunts were not recommended for patients with this deformity [9].

In 1998, Welling et al. reported the results of LVA occlusion in a prospective study in 10 patients with progressive hearing loss. In order to perform LVA occlusion, a fascia graft was inserted between the dura mater of the posterior fossa covering the endolymphatic sac and the aqueduct and the posterior semicircular canal; particular attention was given to avoid opening the endolymphatic sac. In these patients, no statistically significant changes in the rate of hearing loss were identified [8]. In addition, when comparing the hearing results of patients who underwent LVA occlusion and those who did not, no benefit was found when comparing them when considering the natural history of the disease. Based upon this experience, the extraluminal soft-tissue occlusion of the LVA for the purpose of hearing stabilization has not yet been revealed to be significantly effective in changing sensorineural hearing loss accompanying LVA syndrome [8]. However, our EDB technique shows an improvement in hearing in pediatric patients as well as dizziness improvement after a long period of preoperative symptoms. Even though 18% of patients develop worsening hearing after EDB for LVA, hearing improvement occurred in four and hearing stability occurred in one of our six pediatric patients; in addition, hearing stability occurred in four of our five adult patients. While hearing loss was noted preoperatively, hearing remained stable at 5.6 years after surgery. Endolymphatic duct blockage is the first described technique that could be offered to LVA symptomatic patients.

Based on our success rate of endolymphatic duct blockage for refractory Ménière’s disease, we extrapolate that this procedure could be used for the treatment of LVA. In our preliminary report of these eleven patients, vestibular symptoms were remarkably decreased after the blockage of the endolymphatic duct. The improvement was slightly progressive for all patients. A significant improvement was noticed by the fourth month after surgery.

Even though we did not take any measurements of the endolymphatic sac membrane thickness or the endolymph pressure in the sac, we found intraoperatively that there was a significant difference between the endolymphatic sac of the adults and the children who were operated on for LVA. The major difference was found in the sac membrane, which was thicker and more resistant in the adult population than in the children, where the membrane was thin and fragile. We noticed a high endolymphatic pressure in the sac, especially in the pediatric population, in which a large, bulging endolymphatic sac was observed. The aforementioned characteristics of the endolymphatic sac in pediatric patients with LVA lead to a higher risk of sac explosion when performing EDB in children.

Most patients in our study reported dizziness without rotatory vertigo attacks. Dizziness was manifested through head movements and increases in physical activity. Patients reported sensations of floating, unsteadiness and lightheadedness or false sensations of motion. It is reported in the literature that adults’ vestibular symptoms include infrequent vertigo and instability, whereas 30% of children with LVA described incoordination and imbalance [9,18,19,20]. Vestibular symptoms are much more difficult to identify at a young age, although they may appear early.

In LVA pathology, doctors and researchers have generally paid more attention to hearing level than to the vestibular system. However, there has recently been greater awareness of the impacts on vestibular dysfunction.

Song et al. reported in 2018 a unique finding, namely the high incidence of BPPV (18.2%) in LVA subjects. Additionally, three of four BPPV patients complained of simultaneous decreases in hearing. Hearing loss worsening or the displacement of the otoconia from the utricle can occur for a variety of reasons, such as an aggravation of the chemical imbalance or a sudden increase in pressure in the vestibulocochlear system. Thus, secondary BPPV due to the reflux of endolymphatic sac contents is possible [21,22].

It is still unclear why a patient with LVA who suffered from hearing loss in childhood would not develop vestibular symptoms until late in adulthood.

All patients in our study presented with mixed hearing loss with ABG mainly affecting low frequencies. The air–bone gap can be explained by the third window effect. Conductive hearing loss with LVA may occur due to increased endolymphatic pressure. This pressure decreases stapes mobility at the oval window; therefore, sound waves are not transferred well from the middle ear to the cochlea in the inner ear. Merchant et al. suggested the explanation of the air–bone gap in these patients by two mechanisms: shunting air-conducted sound away from the cochlea, thus raising air conduction thresholds, and increasing the difference in impedance between the scala vestibuli side and the scala tympani side of the cochlear partition during bone conduction testing, thus improving thresholds for bone-conducted sound [23].

Even though patients with LVA can benefit from cochlear implant surgery, 82% of patients in our study showed a stable or improved hearing level when comparing the average of hearing loss before and after surgery. These promising results might suggest a blockage of the endolymphatic duct in patients with LVA as a possible treatment to prevent the further damage of the cochlea and to make the cochlea and the vestibule less vulnerable to a sudden increase in cerebrospinal fluid pressure.

The partial blockage of the endolymphatic duct can restore it to a normal size. Therefore, the partial blockage of the enlarged vestibular duct for anatomical reasons, in some cases, may be enough to resolve vestibular symptoms and stabilize hearing loss, although the risk of hearing loss exists. Our two cases of partial EDB maintained stable hearing at the last follow-up. A larger study is needed in order to obtain a valid conclusion on this issue. In addition, we noticed vestibular aqueduct sizes that were larger than 3 mm and 5 mm at the midpoint and at the operculum, respectively, in a patient who had a partial EDB.

In our experience, patients with LVA might be asymptomatic with constant hearing loss over a long period of time. In this case, observation with an annual hearing test is recommended. However, when hearing begins to diminish or when dizziness or vertigo becomes severe, EDB could be offered to these patients.

The blockage of the endolymphatic duct in LVA patients may regulate the pressure variation of the inner ear—therefore preventing hearing loss and dizziness.

To be clinically meaningful, results must be relevant. Controlled trials to validate safety and efficacy for dizziness control and hearing loss preservation and or improvement is needed.

According to our results, a study of downstream physiological impacts using an animal model could be performed.

## 5. Limitations

The sample size is too small and variable to be appropriate for the determination of a true intervention effect. Since the inclusion criteria are too limited, it took 7 years to include 11 cases. We preferred to report this technique and to share it with the otolaryngologist community in order to prepare for a future multicentric study.

This study does not have an appropriate control group and a sham surgery control group would not be ethically possible to rule out a placebo effect. On the other hand, the pure-tone average of air conduction and the mean air–bone gap improved from 63 dB and 20 dB to 42 dB and 16 dB, respectively, in four out of the five pediatric patients and remained stable until the last follow-up visit at 5.6 years post operation, thus increasing the positive effect of the EDB procedure on hearing.

Long-term follow-up and larger studies are certainly required to assess the efficacity of EDB in the management of LVA symptoms and to evaluate the validity of our results. In our study, the patients showed a complete resolution of their vestibular symptoms with a 20% detrimental effect on their hearing levels. Thus, this technique could be considered not only for patients suffering from vestibular symptoms but also to prevent further cochlear damage from the LVA.

## 6. Conclusions

There is no published trial to support the effectiveness of steroids in the treatment of sudden hearing loss associated with LVA. The surgical shunting of the endolymphatic sac is destructive and is not considered to be a treatment option. The extraluminal soft-tissue occlusion of the LVA did not show effective results in altering the sensorineural hearing loss accompanying LVA syndrome. However, endolymphatic duct blockage using two titanium clips seems to be helpful for controlling vestibular symptoms, to stabilize hearing or even to improve hearing in 82% of cases. Nevertheless, there is a risk of hearing worsening, especially in children.

## Figures and Tables

**Table 1:** Shows the demographic data, vestibular symptoms from the clinical history, hearing loss confirmed by audiogram, associated disease and, through an axial high-resolution computed tomography scan, the size of the operated enlarged vestibular aqueduct (VA) at the midpoint and the operculum as well (F: female; M: male).

Patient	Operated Side	Age at Surgery (Years)	Gender	Vestibular Symptoms	Hearing Loss	Associated Disease	Size of VA at Midpoint (mm)	Size of VA at Operculum (mm)
**1**	Right	60	F	+	+	-	2.24	3.65
**2**	Right	38.2	F	+	+	-	2.91	4.01
**3**	Left	30.4	M	+	+	-	4.26	6.46
**4**	Right	19.1	M	+	+	-	2.34	2.85
**5**	Left	41	F	+	+	-	2.38	6.16
**6**	Left	14.8	M	+	+	Trisomy 21	2.52	3.49
**7**	Right	8	F	-	+	-	2.33	3.54
**8**	Left	7.9	F	-	+	-	1.97	4.76
**9**	Right	10.1	M	-	+	-	2.97	3.42
**10**	left	9.7	F	-	+	-	3.28	5.09
**11**	Right	11.3	F	-	+	-	2.96	3.75

**Table 2:** Shows the hearing results before the operation and after the operation at 12 months.

	Bone Conduction		Air Conduction	
	Preoperative	Postoperative	Preoperative	Postoperative
**Adults**	31.75 ± 23	33.75 ± 21.96	42.5 ± 19.68	41.25 ± 19.23
	*p* = 0.45		*p* = 0.43	
**Children**	37.5 ± 16.8	22.5 ± 10.84	58.75 ± 12.55	40 ± 8.36
	*p* = 0.04		*p* = 0.007	

## Data Availability

Data available on request due to restrictions (ethical).

## Supplementary Materials

The following supporting information can be downloaded at: https://www.mdpi.com/article/10.3390/audiolres14020027/s1. Table S1 describes the details of the dizziness handicap inventory (DHI) questionnaire and the method of scoring.; Table S2 describes the details of the dizziness handicap inventory—patient caregiver (DHI-PC) questionnaire and the method of scoring.
