# Supplementary material for: Large Vestibular Aqueduct-Associated Symptoms: Endolymphatic Duct Blockage as a Surgical Treatment
Source: Audiol Res. 2024 Mar 18;14(2):304–16. doi: 10.3390/audiolres14020027 (PMC10961693; doi:10.3390/audiolres14020027)
Supplement: Supplementary file 1 [file audiolres-14-00027-s001.zip › audiolres-2838702-SI.pdf]

Table S1:

The Dizziness Handicap Inventory (DHI)

P1. Does looking up increase your problem?	o Yes<br>o Sometimes<br>o No
E2. Because of your problem, do you feel frustrated?	o Yes<br>o Sometimes<br>o No
F3. Because of your problem, do you restrict your travel for business or recreation?	o Yes<br>o Sometimes<br>o No
P4. Does walking down the aisle of a supermarket increase your problems?	o Yes<br>o Sometimes<br>o No
F5. Because of your problem, do you have difficulty getting into or out of bed?	o Yes<br>o Sometimes<br>o No
F6. Does your problem significantly restrict your participation in social activities, such as going out to dinner, going to the movies, dancing, or going to parties?	o Yes<br>o Sometimes<br>o No
F7. Because of your problem, do you have difficulty reading?	o Yes<br>o Sometimes<br>o No
P8. Does performing more ambitious activities such as sports, dancing, household chores (sweeping or putting dishes away) increase your problems?	o Yes<br>o Sometimes<br>o No
E9. Because of your problem, are you afraid to leave your home without having someone accompany you?	o Yes<br>o Sometimes<br>o No
E10. Because of your problem have you been embarrassed in front of others?	o Yes<br>o Sometimes<br>o No
P11. Do quick movements of your head increase your problem?	o Yes<br>o Sometimes<br>o No
F12. Because of your problem, do you avoid heights?	o Yes<br>o Sometimes<br>o No
P13. Does turning over in bed increase your problem?	o Yes<br>o Sometimes<br>o No
F14. Because of your problem, is it difficult for you to do strenuous homework or yard work?	o Yes<br>o Sometimes<br>o No
E15. Because of your problem, are you afraid people may think you are intoxicated?	o Yes<br>o Sometimes<br>o No
F16. Because of your problem, is it difficult for you to go for a walk by yourself?	o Yes<br>o Sometimes<br>o No
P17. Does walking down a sidewalk increase your problem?	o Yes<br>o Sometimes<br>o No
E18.Because of your problem, is it difficult for you to concentrate	o Yes<br>o Sometimes<br>o No
F19. Because of your problem, is it difficult for you to walk around your house in the dark?	o Yes<br>o Sometimes<br>o No

Table S2:

NAME: DAT E:

VANDERBILT PEDIATRIC DIZZINESS HANDICAP INVENTORY (DHI)
(Age 5-12)

Instructions: The purpose of this questionnaire is to identify difficulties that your child may be experiencing because of his or her dizziness or unsteadiness. Please answer “yes”, “no”, or “sometimes” to each question. **Answer each question as it pertains to your child’s dizziness problem only**.

	Yes (4)	Sometimes (2)	No (0)
1. Does your child’s problem make him/her feel tired?			
2. Is your child’s life ruled by his/her problem?			
3. Does your child’s problem make it difficult for him/her to play?			
4. Because of his/her problem, does your child feel frustrated?			
5. Because of his/her problem, has your child been embarrassed in front of others?			
6. Because of his/her problem, is it difficult for your child to concentrate?			
7. Because of his/her problem, is your child tense?			
8. Do other people seem irritated with your child’s problem?			
9. Because of his/her problem, does your child worry?			
10. Because of his/her problem, does your child feel angry?			
11. Because of his/her problem, does your child feel “down”?			
12. Because of his/her problem, does your child feel unhappy?			
13. Because of his/her problem, does your child feel different from other children?			
14. Does your child’s problem significantly restrict his/her participation in social or educational activities, such as going to dinner, meeting with friends, field trips, or to parties?			
15. Because of your child’s problem, is it difficult for him/her to walk around the house in the dark?			
16. Because of his/her problem, does your child have difficulty walking up stairs?			
17. Because of his/her problem, does your child have difficulty walking one or two blocks?			
18. Because of his/her problem, does your child have difficulty riding a bike or scooter?			
19. Because of his/her problem, does your child have difficulty reading or doing schoolwork?			
20. Does your child's problem make it difficult to successfully do activities that others his/her age can do?			
21. Because of his/her problem, does your child have trouble concentrating at school?			
		**TOTAL SCORE**	

McCaslin DL, Jacobson GP, Lambert W, English LN, Kemph AJ (2015). “The development of the vanderbilt pediatric dizziness handicap inventory for patient caregivers (DHI-PC).” *Int J Pediatr Otorhinolaryngol.* 79 (10): p1662-1666. doi: 10.1016/j.ijporl.2015.07.017

Significant change: >18 points; 0-16 no activity limitation; 16-26 mild activity limitation; 26-43 moderate activity limitation; >43 severe limitation
